# RNA interference: more than a research tool in the vertebrates' adaptive immunity

**DOI:** 10.1186/1742-4690-2-35

**Published:** 2005-05-25

**Authors:** Johnson Mak

**Affiliations:** 1 Virology Program, Macfarlane Burnet Institute for Medical Research and Public Health, Melbourne, Australia; 2 Department of Biochemistry and Molecular Biology, Monash University, Clayton, Australia

**Keywords:** RNA silencing, siRNA, miRNA, HIV, PFV-1, vertebrate, immunity, viral invasion

## Abstract

In recent years, RNA silencing, usage of small double stranded RNAs of ~21 – 25 base pairs to regulate gene expression, has emerged as a powerful research tool to dissect the role of unknown host cell factors in this 'post-genomic' era. While the molecular mechanism of RNA silencing has not been precisely defined, the revelation that small RNA molecules are equipped with this regulatory function has transformed our thinking on the role of RNA in many facets of biology, illustrating the complexity and the dynamic interplay of cellular regulation. As plants and invertebrates lack the protein-based adaptive immunity that are found in jawed vertebrates, the ability of RNA silencing to shut down gene expression in a sequence-specific manner offers an explanation of how these organisms counteract pathogen invasions into host cells. It has been proposed that this type of RNA-mediated defence mechanism is an ancient form of immunity to offset the transgene-, transposon- and virus-mediated attack. However, whether 1) RNA silencing is a natural immune response in vertebrates to suppress pathogen invasion; or 2) vertebrate cells have evolved to counteract invasion in a 'RNA silencing' independent manner remains to be determined. A number of recent reports have provided tantalizing clues to support the view that RNA silencing functions as a physiological response to regulate viral infection in vertebrate cells. Amongst these, two manuscripts that are published in recent issues of *Science *and *Immunity*, respectively, have provided some of the first direct evidences that RNA silencing is an important component of antiviral defence in vertebrate cells. In addition to demonstrating RNA silencing to be critical to vertebrate innate immunity, these studies also highlight the potential of utilising virus-infection systems as models to refine our understanding on the molecular determinants of RNA silencing in vertebrate cells.

## 

RNA silencing was originally recognised as post-transcriptional gene silencing in plants (PTGS) [1], co-suppression in plants [2], or RNA-mediated virus resistance in plants [3]. It was subsequently understood that a similar mechanism (RNA interference) is also found in *Caenorhabditis elegans *[4] and fungi [5,6]. The generation of ~25 nucleotide RNA which pair to yield a ~19 base-pair double helix is the common denominator amongst these different systems. These RNAs are able to 'silence' the target mRNA through complementarity, which can leads to the degradation of the target mRNA or suppression of protein translation from the target mRNA.

When these small RNAs are generated from double-stranded RNA, they are known as small interfering RNAs (siRNAs); however, if these small RNAs are produced from within the cell as natural RNAs that fold into imperfect hairpin structures, they are referred to as microRNAs (miRNAs). siRNAs are produced by cleavage mediated through a ribonuclease-III (RNaseIII)-related enzyme known as Dicer, which gives rise to the siRNA duplex. This siRNA duplex is then unwound by RNA helicase and assembled into the RNA induced silencing complex (RISC). It is believed that the RISC then directs the siRNA to the target mRNA via sequence specificity which leads to cleavage of the target mRNA and silencing of the target gene. In contrast with siRNAs, miRNAs are first produced as ~70 nucleotides pri-microRNAs (pri-miRNA) in the nucleus, which are then cleaved by a RNaseIII-like enzyme known as Drosha to generate the pre-miRNA. The pre-miRNA is then transported into the cytoplasm aided by Exportin 5. Once the pre-miRNA has reached the cytoplasm, Dicer cleaves the pre-miRNA to generate the miRNA duplex. The miRNA duplex is subsequently unwound by RNA helicase and assembles with RISC. As seen with siRNA, some of the miRNA will be guided by RISC for cleavage and degradation of the target mRNA, however, most miRNA will suppress translation of the target mRNA by binding to the 3' untranslated region (3'UTR) of the target.

Because plants and invertebrates lack the protein-based adaptive immunity that is found in higher vertebrates, it is believed that the sequence-specific RNA silencing mechanism is important for the host cell to fight off invading-viruses, -pathogens and -nucleic acids [7]. Similar to the protein-based adaptive immune response in higher vertebrates, the RNA silencing in plant and *C elegans *can be spread to other uninfected cells in the organism to prevent further infection. This spreading of RNA silencing relies on an RNA-directed RNA polymerase (RdRP) to amplify the RNA silencing targeting sequences. Interestingly, no RdRP was identified in either *Drosophila melanogaster *or human genomes when a BLAST search was performed on the nearly completed genomes for these two species [8]. The lack of RdRP in *Drosophila melanogaster *and human genomes to amplify the siRNA signals as part of their immune responses could suggest that the existence of RNA silencing machineries in these species represents a 'molecular fossil' of an ancient innate immunity, and both *Drosophila *and vertebrates have since evolved to counter viral invasion through an RNA silencing independent mechanism.

Recent studies have provided a number of indirect and tantalising clues to support the participation of RNA silencing in viral infection of vertebrates. Using a heterologous system, it has been shown that some of the mammalian virus-encoded proteins, such as influenza viral protein NS1 and vaccinia viral protein E3L, have a negative regulatory role on RNA silencing in both plant and insect cells, providing circumstantial evidence to illustrate the potential involvement of RNA silencing of these mammalian viral proteins in their natural vertebrate target hosts [9-11]. Others have shown that the VA non-coding RNAs of adenovirus can down regulate RNA silencing in mammalian cells [12]. However, one must bear in mind that the double strand nature of the siRNAs can results in an interferon-mediated activation of the JAK/STAT (Janus kinase/signal transducer and activator of transcription) pathway and global up-regulation of interferon-stimulated genes. This process is regulated in part by the dsRNA-dependent protein kinase (PKR). As the disruption of adenoviral VA1 RNA significantly affects the level of adenovirus found in infected cells through a strong activation of PKR activity, making it difficult to isolate the precise contribution of the adenoviral VA1 non-coding RNA to the process RNA silencing in mammalian cells [12]. Similarly, using herpesviruses infection systems, it was found that a number of herpesviruses (such as Epstein-Barr virus, Kaposi sarcoma-associated virus, human cytomegalovirus and mouse gammaherpesvirus 68) encode an array of miRNA genes [13-15], however, the physiological functional significance of these miRNAs are yet to be validated [13-15].

Direct evidence of the importance of RNA silencing in vertebrates to control viral invasion has recently emerged from studies using human retroviruses. In the first study, primate foamy virus type 1 (PFV-1) was used to infect mammalian cells [16]. While *Lecellier et al. *were unable to identify viral derived small RNA that suppressed the propagation of PFV-1 in the host cell, they noted that PFV-1 infection promoted the non-specific accumulation of cellular derived miRNAs as a means to interfere with the miRNA regulatory pathway [16], a situation that has been previously described in plant virus infection. More specifically, they have reported the presence of a cellular derived miRNA that can effectively suppressed PFV-1 replication [16]. In contrast to the PFV-1 study, *Bennasser et al. *found that human immunodeficiency virus type 1 (HIV-1) contains a rare siRNA precursor within its genomes, which can be utilised by the host cell to regulate HIV-1 infection [17]. It is not excluded that similar to the PFV-1 system, other yet to be identified host cell sequence derived miRNA may also play roles in suppressing HIV-1 infection. One remarkable commonality between PFV-1 and HIV-1 is that both viruses have evolved to use their respective viral transcriptional factors (the PFV-1 Tas and the HIV-1 Tat protein, respectively) as suppressors of RNA silencing (SRS) to counteract this host cell immune response [16,17]. Using a transcriptional inactive HIV-1 Tat mutant, *Bennasser et al. *further demonstrated that the transcriptional activity of Tat is not essential for this SRS activity of HIV-1 Tat [17]. Currently, it remains unclear whether the transcriptional activity of PFV-1 Tas is also dispensable for its SRS function. Further study is required to unravel the precise mechanism by PFV-1 Tas and HIV-1 Tat indict the host cell's antiviral RNA silencing. It remains to be seen whether these two viruses share a similar mechanism in this type of innate immunity, the demonstration of RNA silencing in vertebrate cells clearly highlights the significance of this ancient immunity in higher eukaryotes [16,17]. This is further underscored by the rarity of siRNA sequence found within the HIV-1 genome and the lack of siRNA precursor sequence in PFV genome, implying that these two viruses have evolved under the selective pressure of RNA silencing and have attempted to alter their sequences to evade this antiviral selection. These observations also emphasise the potential to explore RNA silencing as means to suppress viral infection (such as HIV-1) in mammalian cells, although an effective strategy to deliver small interference RNAs into the target cells has to be developed. On a separate note, the recent studies have shown that viral infection of vertebrate cells can be used as an important tool to dissect the molecular basis for this fascinating but somewhat 'poorly defined' silencing process in mammals.

## Competing interests

The author(s) declare that they have no competing interests.

**Figure 1:**
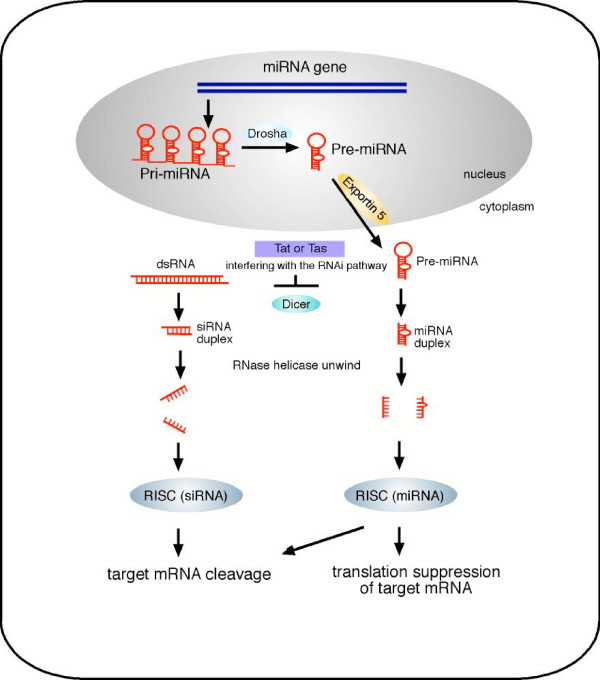
***Model of RNA silencing pathway***. The biogenesis of RNA silencing transcripts can be derived from either the host cell nucleus mRNA pathway to yield miRNA or the cytoplasmic double strand RNA to yield siRNA. HIV-1 and PFV have evolved to use their transcriptional factor to counteract this ancient host cell immunity.
